# The moderating role of social support in the relationship between alexithymia and problematic smartphone use among Chinese depressed adolescents: a cross-sectional study

**DOI:** 10.3389/fpsyt.2025.1650290

**Published:** 2025-10-01

**Authors:** Shumiao Liu, Yufeng Wu, Fengxia Sun, Yongjie Zhou, Aihua Yin

**Affiliations:** ^1^ Department of Psychiatry, Shandong Mental Health Center, Shandong University, Jinan, China; ^2^ Shenzhen Mental Health Center, Shenzhen Kangning Hospital, Shenzhen, China

**Keywords:** alexithymia, problematic smartphone use, social support, depressed adolescents, moderating effect

## Abstract

**Background:**

Alexithymia is closely related to problematic smartphone use (PSU) in adolescents, but its mechanism in adolescents with depression is still unclear. The aim of this study was to investigate the predictive effect of alexithymia on PSU in depressed adolescents and to examine the moderating effect of social support (family, friends, significant others) on this relationship.

**Methods:**

A total of 2343 adolescents with depressive disorder aged 12–18 years from 14 medical institutions in China were included in this cross-sectional study. The Toronto Alexithymia Scale, Mobile Phone Addiction Index and Multidimensional Perceived Social Support Scale were used to evaluate the core variables. Hierarchical regression analysis was used to test the moderating effect after controlling demographic variables.

**Results:**

Alexithymia was significantly and positively associated with PSU (r = 0.343, p < 0.01), with Difficulty in Recognizing Feelings having the strongest association (r = 0.348). Stratified regression revealed that family support (B = -0.625, p = 0.005) and friend support (B = -0.577, p = 0.013) significantly attenuated the positive predictive effect of alexithymia on PSU, whereas there was no significant moderating effect of significant others support.

**Conclusion:**

Research shows that social support mitigates the risk of (PSU) among depressed adolescents with alexithymia, with family and friend support as key protective factors. These findings highlight the need for clinical screening and interventions targeting adolescents with alexithymia and low social support, who are at high risk for PSU. Integrating family systems therapy and friend support programs into clinical interventions may enhance emotional regulation and reduce smartphone dependence, thereby improving depressive symptoms.

## 1 Introduction

Smartphones have become indispensable tools for communication and information access in modern life, with their rich entertainment and social functions significantly enhancing convenience. However, adolescents’ relatively weaker self-control makes smartphones highly attractive to them, often leading to Problematic Smartphone Use (PSU) (1). Data indicates that a high percentage (93.9%) of Chinese adolescents access the internet via smartphones (2), raising concerns about excessive use. PSU refers to the excessive (in time or frequency) or inappropriate use of smartphones, characterized by the individual’s difficulty in self-control despite awareness of negative consequences. This behavior results in impairments to physical and mental health, academic performance, and daily life, manifesting as withdrawal symptoms akin to addiction (3–5). Although there is ongoing debate within academia about classifying PSU as a mental disorder (6–8), it shares similarities with internet addiction (9). Therefore, this study draws upon the “internet addiction” research framework, focusing on the smartphone use behavior itself without directly associating it with a mental disorder diagnosis.

The negative impact of PSU is particularly pronounced among adolescents with depressive disorders. Due to their immature psychosocial development and personality formation, adolescents are more vulnerable to adverse psychological consequences from PSU (10). Research demonstrates that PSU may exacerbate symptoms of anxiety and depression (11), and even increase the risk of self-harm or suicide (12, 13). It is also associated with attention deficits, hyperactivity, and impulsivity (14), as well as academic impairment (15). At the family level, excessive smartphone use correlates with increased parent-child conflict and decreased family cohesion (16). Regarding physical health, PSU may lead to sleep disturbances and vision problems (17, 18). Furthermore, excessive social media use among adolescents has been confirmed to be associated with mood disorders (19). Additionally, the phenomenon of social media intervention has been explored, with evidence suggesting that targeted interventions can mitigate some of the negative impacts of excessive use on adolescents’ mental health (20). Moreover, adolescent anhedonia—the inability to derive pleasure from normally enjoyable activities—has been identified as a significant factor that increases susceptibility to PSU, as adolescents with this condition may turn to the Internet for instant gratification and emotional escape (21). Similarly, the relationship between loneliness, Internet use, and depression has been widely studied, with findings indicating that excessive online engagement, especially on social media, contributes to feelings of isolation and worsens depressive symptoms (22).Consequently, PSU likely exacerbates the challenges faced by adolescents with depressive disorders, necessitating in-depth investigation into its underlying psychological mechanisms.

Alexithymia, regarded as a personality trait, involves difficulties in identifying, describing, and regulating emotions (23). This includes challenges in distinguishing emotions from physical sensations, limitations in verbalizing emotional experiences, and restricted imaginative thought (24, 25). For adolescents, alexithymia impedes the expression and understanding of negative emotions, potentially leading to the accumulation and worsening of depressive symptoms, prolonged episodes, and increased relapse risk (26). It also elevates the likelihood of suicidal ideation and non-suicidal self-injury (27, 28). Studies have confirmed a significant correlation between the severity of alexithymia and PSU, identifying it as a risk factor for overuse (29). Social support, defined as the emotional and material assistance obtained from social networks (30), primarily refers to informal support from family, relatives, and friends in this study. Adolescents perceiving lower levels of social support are more likely to exhibit problematic use (31), possibly because they attempt to seek emotional fulfillment and social connection through their phones. Multiple studies consistently find that inadequate social support exacerbates negative emotions and loneliness, weakens self-regulation and self-control, and increases the risk of internet addiction (32, 33). A meta-analysis specifically focusing on Chinese adolescents (incorporating 92 studies with 59,716 participants) also clearly demonstrates a significant negative correlation between mobile phone addiction and social support (34). Therefore, social support, as a resource for coping with stress, may play a crucial role in mitigating the negative effects of alexithymia and PSU among adolescents with depressive disorders.

Although existing research indicates an association between alexithymia and PSU, the moderating role of social support in this relationship has not been systematically studied among adolescents with depressive disorders. Given the negative consequences associated with both alexithymia and PSU, it is crucial to clarify the psychological mechanisms underlying their interplay in order to understand their interaction and develop interventions aimed at reducing PSU. The primary aim of this study was to examine whether alexithymia predicts PSU, while the secondary aim was to explore whether social support moderates this relationship. This research aims to provide a theoretical foundation for understanding the factors influencing the mental health of adolescents with depressive disorders and to inform the development of relevant interventions.

## 2 Materials and methods

### 2.1 Study design and population

The present study was of a cross-sectional study. Participants were adolescents diagnosed with depressive disorders. These individuals were recruited from the psychiatric outpatient departments of 14 mental health institutions or general hospitals across nine provinces in China. The study recruitment period commenced in December 2020 and ended in December 2021. Inclusion criteria were as follows: (i) Aged 12–18 years; (ii) Diagnosed with a depressive disorder (MDD, BD-D, or depressive state) according to DSM-5 criteria by senior psychiatrists; (iii) Adequate cognitive ability to complete the research questionnaire; (iv) Written informed consent was obtained from both the participant and their legal guardian. Exclusion criteria included: (i) Diagnosis of pervasive developmental disorders or intellectual disability; (ii) Presence of suicidal or homicidal ideation requiring immediate intervention; (iii) Active psychotic symptoms, such as schizophrenia or a manic episode. This study was reviewed and approved by the Ethics Committee of Shenzhen Kangning Hospital (IRB: 2020-k021-02). Prior to the commencement of the survey, parents of each adolescent provided written informed consent for their children to take part in this study. This study followed the Strengthening the Reporting of Observational Studies in Epidemiology (STROBE) checklist for cross-sectional research (35) (see supplementary material).

### 2.2 Assessment of mobile phone addiction index (Chinese version)

The degree of problematic smartphone use was assessed using the Chinese version of the Mobile Phone Addiction Index (MPAI) (36). This scale consists of 17 self-reported items designed to evaluate smartphone usage, covering four subscales: uncontrollability, withdrawal, escape, and inefficiency. Each item is rated on a 5-point scale (1 = never, 5 = frequently), with a total score ranging from 17 to 85. Higher scores indicate a greater degree of smartphone dependence, and a score of ≥51 is defined as problematic smartphone use. In this study, the Cronbach’s α for the scale was 0.80, indicating good reliability.

### 2.3 Assessment of alexithymia

This study utilized the Toronto Alexithymia Scale (TAS) to assess alexithymia (37). The 20-item Toronto Alexithymia Scale (TAS-20) was used to assess the severity of alexithymia. The scale comprises three subdimensions: Difficulty Identifying Feelings (DIF, 7 items), Difficulty Describing Feelings (DDF, 5 items), and Externally-Oriented Thinking (EOT, 8 items). Each item is rated on a 5-point Likert scale ranging from 1 (strongly disagree) to 5 (strongly agree), resulting in a total score between 20 and 100. Higher scores indicate greater levels of alexithymia. According to established cut-off criteria, scores of 51 or below are classified as “no alexithymia”, scores between 52 and 60 as “possible alexithymia”, and scores of 61 or above as “alexithymia”. In the present study, the TAS-20 demonstrated good internal consistency, with a Cronbach’s alpha of 0.78.

### 2.4 Assessment of social support

This study employed the Multidimensional Scale of Perceived Social Support (MSPSS) to evaluate participants’ perceived social support (38). The scale consists of 12 self-reported items designed to measure the extent of social support received from family, friends, and significant others. An example item is: “There is a special person who is always around when I need them.” Each item is rated on a 7-point Likert scale, ranging from 1 (very strongly disagree) to 7 (very strongly agree). The scores are used to calculate three subscale scores (family, friends, and significant others) and an overall score. The subscale and total scores are expressed as the average of the respective items, with higher scores indicating higher levels of perceived social support. In this study, the Cronbach’s α for the MSPSS was 0.84, indicating strong internal consistency.

### 2.5 Covariates

To control for potential confounding factors, the following covariates were included in the analyses: demographic characteristics (gender, age, only-child status, and residential area), parental marital status, parental highest level of education, quality of the parent–child relationship, and history of self-injurious behavior.

### 2.6 Data analysis

The collected data underwent preliminary screening to remove samples with obvious outliers. Normality tests were performed for all variables. Descriptive statistical analyses were conducted to calculate the means, standard deviations, minimum, and maximum values for alexithymia, problematic smartphone use, and social support. Pearson correlation coefficients were used to analyze the pairwise correlations among alexithymia, problematic smartphone use, and social support. To test the moderating role of social support in the relationship between alexithymia and problematic smartphone use, hierarchical regression analysis was employed. The analysis followed these steps: (1) Enter control variables (e.g., age, gender, family structure) into the model. (2) Add the main effects of alexithymia and social support (family support, friend support, and significant other support). (3) Introduce the interaction term between alexithymia and social support to examine the moderating effect of social support.

A simple slope analysis was conducted to further explore the strength of the relationship between alexithymia and problematic smartphone use at different levels of social support. An interaction plot was created to visually illustrate the moderating effect of social support. All statistical tests were two-tailed, and the significance level was set at 0.05. Results with p-values < 0.05 were considered statistically significant.

## 3 Results

### 3.1 Demographic characteristics of the sample

A total of 2,343 adolescents completed the survey. Participants ranged in age from 12 to 18 years (M = 14.99, SD = 1.65), with 517 males (22.1%) and 1,826 females (77.9%). Among them, 684 adolescents (29.2%) were only children, while 1,659 (70.8%) had siblings. Additionally, 67.4% of the participants were from urban areas, and 32.6% were from rural areas. Detailed participant characteristics are shown in **Table 1**.

**Table 1:** Demographic characteristics of the adolescents with depressive disorders.

Variables	n	%
Gender		
Male	517	22.1
Female	1826	77.9
Age		
12	138	5.9
13	368	15.7
14	466	19.9
15	425	18.1
16	446	19.0
17	352	15.0
18	148	6.3
Only child		
Yes	648	29.2
No	1659	70.8
Living area		
Urban	1580	67.4
Rural	763	32.6
Father’s education level		
Primary school or below	301	12.8
Junior high school	882	37.6
High school/vocational high school	527	22.5
College	272	11.6
University	318	13.6
Master’s	38	1.6
Doctorate	5	0.2
Mother’s education level		
Primary school or below	532	22.7
Junior high school	809	34.5
High school	453	19.3
College	283	12.1
University	238	10.2
Master’s	28	1.2
Self-injurious behavior		
Yes, occurred a year ago	182	7.8
Yes, within the past year	1782	76.1
No	379	16.2
Father-child relationship		
Very good	216	9.2
Good	569	24.3
Average	976	41.8
Poor	384	16.4
Very poor	195	8.3
Mother-child relationship		
Very good	315	13.4
Good	866	37.0
Average	819	35.0
Poor	232	9.9
Very poor	111	4.7
Parental marital status		
Normal	1838	78.4
Divorced	247	10.5
Separated	63	2.7
Remarried	132	5.6
Father/mother/parents deceased	63	2.7

### 3.2 Descriptive statistics and correlations among main variables

Descriptive statistics and correlations for measured variables are presented in **Tables 2** and **3**. Using the chi-square test (χ²), the relationship between different levels of alexithymia and problematic smartphone use among adolescents was examined. From the **Table 2**, it can be observed that there is a significant difference in the incidence of problematic smartphone use among adolescents with varying degrees of alexithymia, with a significance level of 0.01 (*χ²* = 80.488, *p* = 0.000 < 0.01). This suggests that alexithymia is significantly associated with problematic smartphone use, particularly among adolescents with alexithymia, where the rate of problematic smartphone use is notably higher. This indicates that alexithymia may be a risk factor for problematic smartphone use.

**Table 2:** Correlation between alexithymia and problematic smartphone use in adolescents with depression.

Variable	Sub-domains	Problematic Smartphone Use (n/%)		Total	*χ^2^*	*P*
		No	Yes			
Alexithymia	No Alexithymia	103(6.71)	13(1.61)	116(4.95)	80.488	0.000^**^
	Possible Alexithymia	312(20.31)	78(9.67)	390(16.65)		
	Alexithymia	1121(72.98)	716(88.72)	1837(78.40)		
Total		1536	807	2343

*p < 0.05, **p < 0.01.

**Table 3:** Pearson correlation coefficients between excessive problematic smartphone use, alexithymia, and social support.

Variable	Mean	SD	1	2	3	4	5	6	7	8	9
1 Problematic Smartphone Use	45.474	13.415	1								
2 Social Support	46.853	16.718	-0.088**	1							
3 Family Support	15.039	6.103	-0.180**	0.736**	1						
4 Friend Support	16.065	6.812	-0.020	0.876**	0.439**	1					
5 Significant Other Support	15.749	7.072	-0.032	0.885**	0.453**	0.729**	1				
6 Alexithymia	67.800	10.127	0.343**	-0.115**	-0.160**	-0.077**	-0.060*	1			
7 Difficulty Identifying Feelings DIF	26.263	6.105	0.348**	-0.342**	-0.358**	-0.275**	-0.236**	0.823**	1		
8 Difficulty Describing Feelings, DDF	16.879	2.914	0.266**	-0.204**	-0.169**	-0.192**	-0.153**	0.787**	0.636**	1	
9 Externally-Oriented Thinking, EOT	24.658	4.767	0.121**	0.318**	0.221**	0.305**	0.268**	0.589**	0.079**	0.247**	1

SD, Standard Deviation *p < 0.05, **p < 0.01.

**Table 3** presents the Pearson correlation coefficients among problematic smartphone use (PSU), alexithymia, and perceived social support. PSU was significantly and positively correlated with overall alexithymia (r = 0.343, p < 0.01), indicating that higher levels of alexithymia are associated with more severe problematic smartphone use. Among the subdimensions of alexithymia, difficulty identifying feelings (DIF) showed the strongest correlation with PSU (r = 0.348, p < 0.01), followed by difficulty describing feelings (DDF; r = 0.266, p < 0.01), and externally-oriented thinking (EOT; r = 0.121, p < 0.01).Perceived social support was negatively correlated with PSU (r = -0.088, p < 0.01). Among the three sources of support, only family support demonstrated a significant negative correlation with PSU (r = -0.180, p < 0.01), whereas correlations with friend support and significant other support were nonsignificant.

### 3.3 Regression analysis and moderating effects of social support

**Figure 1:**
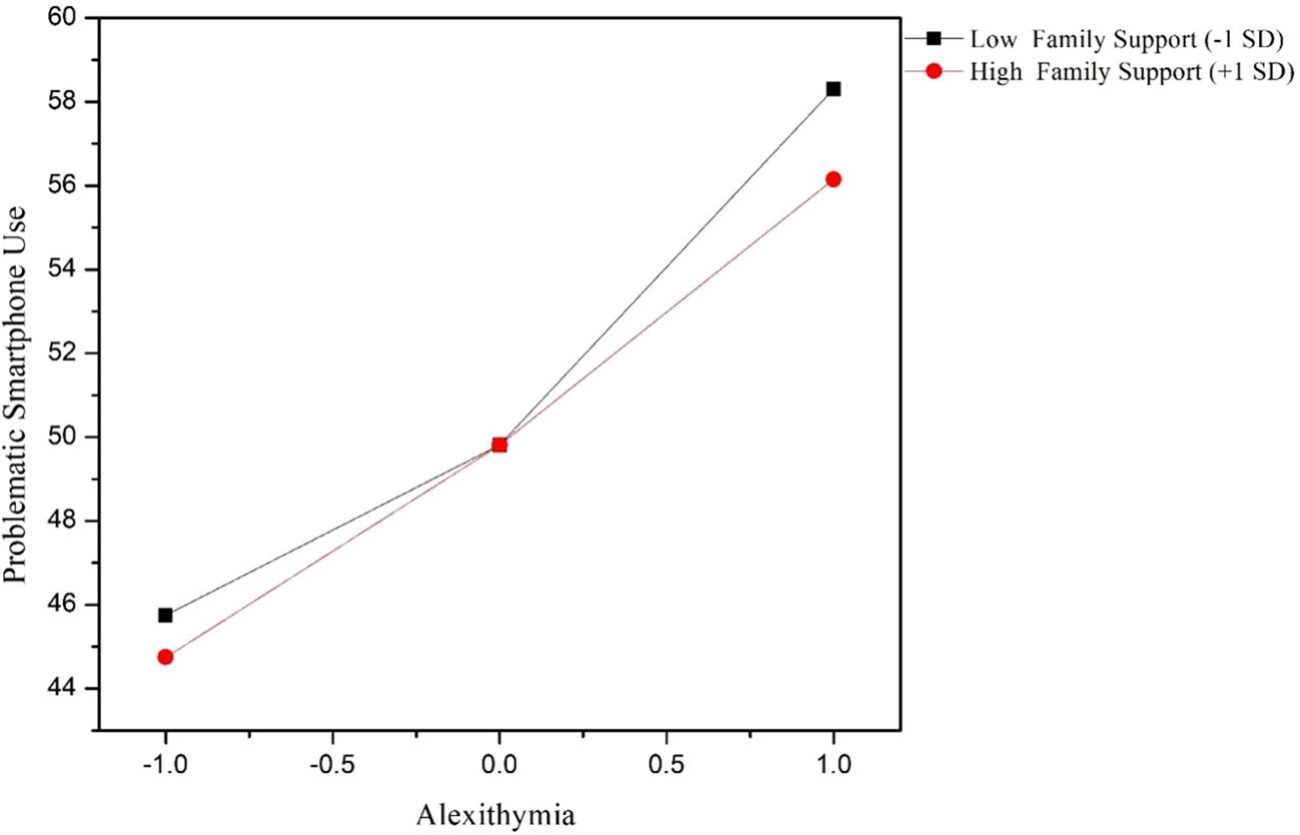
Interaction of alexithymia and family support on problematic smartphone use.

**Figure 2:**
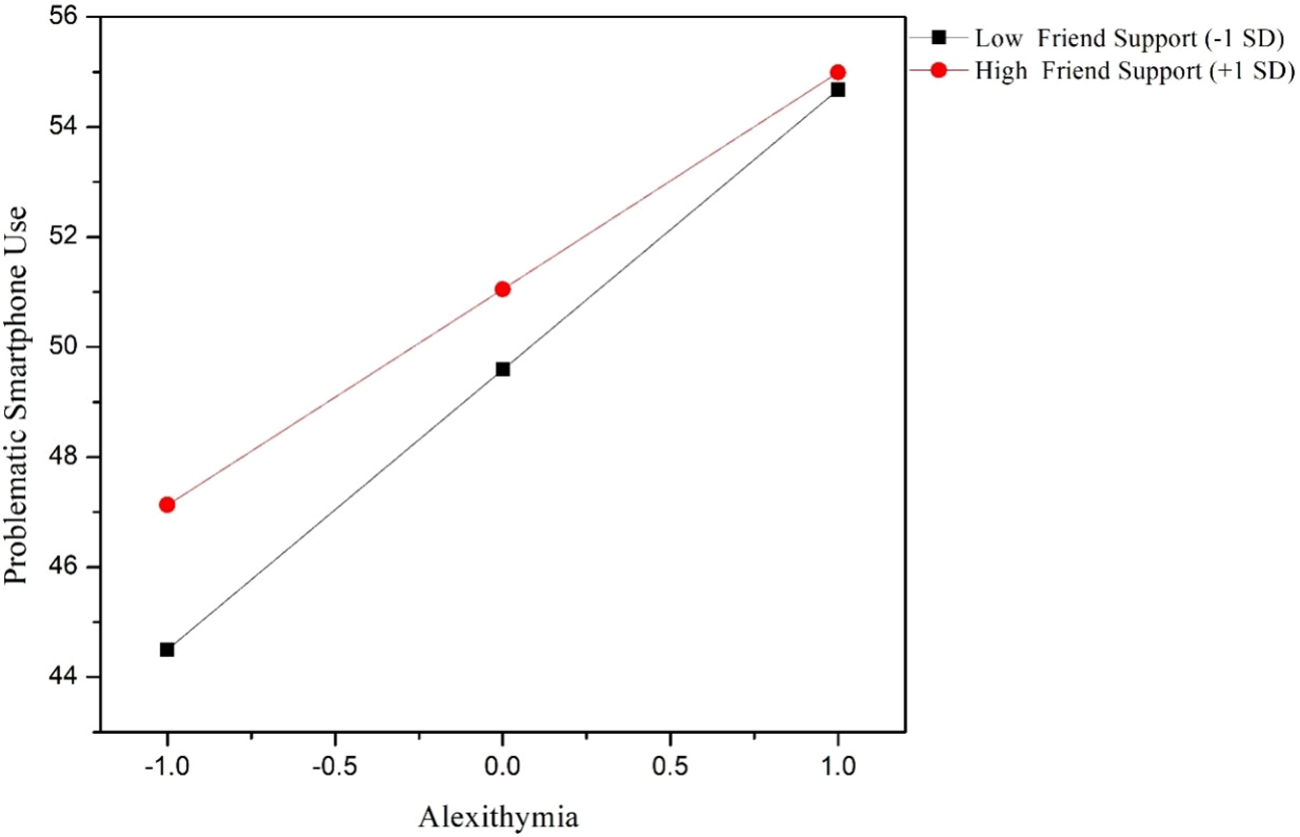
Interaction of alexithymia and friend support on problematic smartphone use.

To further examine whether different types of social support moderated the association between alexithymia and problematic smartphone use, hierarchical regression analyses were conducted separately for family support, friend support, and significant other support. Age and gender were controlled for in all models. In the family support model, alexithymia was found to be a significant positive predictor of problematic smartphone use (B = 4.437, *p* < 0.001). The interaction term between alexithymia and family support was significant and negative (B = -0.625, *p* = 0.005), indicating that higher levels of family support attenuated the positive relationship between alexithymia and problematic smartphone use. In the friend support model, alexithymia again positively predicted problematic smartphone use (B = 4.513, *p* < 0.001). Friend support exhibited a significant negative association with problematic smartphone use (B = 0.724, *p* = 0.007). Moreover, the interaction between alexithymia and friend support was significant (B = -0.577, *p* = 0.013), suggesting that higher friend support weakened the association between alexithymia and problematic smartphone use. In contrast, in the significant other support model, although significant other support independently predicted lower levels of problematic smartphone use (B = 0.568, *p* = 0.033), the interaction between alexithymia and significant other support was not statistically significant (B = -0.250, *p* = 0.285). This indicates that significant other support did not moderate the relationship between alexithymia and problematic smartphone use. Detailed information can be found in **Tables 4**–**6** and **Figures 1**, **2**.

**Table 4:** Moderation effect analysis of alexithymia and family support on problematic smartphone use in adolescents with depression.

Outcome	Model 1				Model 2				Model 3			
	B	SE	T	β	B	SE	t	β	B	SE	t	β
Constant	50.065	3.128	16.006**	–	50.516	3.136	16.108**	–	50.318	3.132	16.064**	–
Age	-0.697	0.159	-4.394**	-0.085	-0.647	0.161	-4.020**	-0.079	-0.636	0.161	-3.959**	-0.078
Gender	-0.569	0.631	-0.901	-0.018	-0.604	0.631	-0.956	-0.019	-0.561	0.631	-0.889	-0.017
Alexithymia	4.529	0.26	17.447**	0.336	4.475	0.261	17.138**	0.332	4.437	0.261	16.994**	0.329
Family support					-0.581	0.318	-1.825	-0.043	-0.506	0.319	-1.588	-0.038
Alexithymia ×family support									-0.625	0.225	-2.779**	-0.053
*R ^2^*	0.145				0.146				0.149			
Adjusted *R ^2^*	0.142				0.143				0.145			
*F*	F (7,2335)=56.343,p=0.000				F (8,2334)=49.766,p=0.000				F (9,2333)=45.222,p=0.000			
*ΔR* ^2^	0.145				0.001				0.003			
*ΔF*	F (7,2335)=56.343,p=0.000				F (1,2334)=3.332,p=0.068				F (1,2333)=7.723,p=0.005			

Dependent variable: Problematic Smartphone Use; Interaction: interaction of Alexithymia and Family Support. *p<0.05, **p<0.01.

**Table 5:** Moderation effect analysis of alexithymia and friend support on problematic smartphone use in adolescents with depression.

Outcome	Model 1				Model 2				Model 3			
	B	SE	t	β	B	SE	t	β	B	SE	t	β
Constant	50.065	3.128	16.006**	–	50.186	3.124	16.065**	–	50.322	3.121	16.124**	–
Age	-0.697	0.159	-4.394**	-0.085	-0.751	0.16	-4.706**	-0.092	-0.748	0.16	-4.692**	-0.092
Gender	-0.569	0.631	-0.901	-0.018	-0.596	0.631	-0.946	-0.018	-0.606	0.63	-0.963	-0.019
Alexithymia	4.529	0.26	17.447**	0.336	4.569	0.26	17.595**	0.339	4.513	0.26	17.336**	0.335
Friend Support					0.724	0.268	2.703**	0.054	0.731	0.268	2.73**	0.054
Alexithymia ×friend Support									-0.577	0.231	-2.497*	-0.048
*R^2^*	0.145				0.147				0.149			
Adjusted *R^2^*	0.142				0.144				0.146			
*F*	F (7,2335)=56.343,p=0.000				F (8,2334)=50.347,p=0.000				F (9,2333)=45.546,p=0.000			
*ΔR* ^2^	0.145				0.003				0.002			
*ΔF*	F (7,2335)=56.343,p=0.000				F (1,2334)=7.308,p=0.007				F (1,2333)=6.237,p=0.013			

Dependent variable: Problematic Smartphone Use; Interaction: interaction of Alexithymia and Friend Support. *p<0.05, **p<0.01.

**Table 6:** Moderation effect analysis of alexithymia and significant other support on problematic smartphone use in adolescents with depression.

Outcome	Model 1				Model 2				Model 3			
	B	SE	t	β	B	SE	t	β	B	SE	t	β
Constant	50.065	3.128	16.006**	–	49.88	3.127	15.954**	–	49.954	3.127	15.97**	–
Age	-0.697	0.159	-4.394**	-0.085	-0.715	0.159	-4.506**	-0.088	-0.715	0.159	-4.502**	-0.087
Gender	-0.569	0.631	-0.901	-0.018	-0.622	0.631	-0.985	-0.019	-0.609	0.631	-0.965	-0.019
Alexithymia	4.529	0.26	17.447**	0.336	4.547	0.26	17.519**	0.337	4.522	0.261	17.35**	0.336
significant other support					0.568	0.267	2.128*	0.042	0.556	0.267	2.082*	0.041
Alexithymia ×significant other support									-0.250	0.234	1.07	-0.021
*R ^2^*	0.145				0.146				0.147			
Adjusted *R ^2^*	0.142				0.143				0.143			
*F*	F (7,2335)=56.343,p=0.000				F (8,2334)=49.941,p=0.000				F (9,2333)=44.521,p=0.000			
*ΔR ^2^*	0.145				0.002				0.001			
*ΔF*	F (7,2335)=56.343,p=0.000				F (1,2334)=4.528,p=0.033				F (1,2333)=1.144,p=0.285			

Dependent variable: Problematic Smartphone Use; Interaction: interaction of Alexithymia and Significant other Support Support. *p<0.05, **p<0.01.

## 4 Discussion

This study aimed to investigate the impact of alexithymia on problematic smartphone use among depressed adolescents and the buffering role of social support in this relationship. The study included 2,343 adolescents recruited from 14 mental health institutions or psychiatric outpatient departments in general hospitals across nine provinces in China. The results and discussion are as follows:

### 4.1 The role of alexithymia in predicting problematic smartphone use

Consistent with our hypotheses, alexithymia was a significant positive predictor of PSU among adolescents with depressive disorders. This finding aligns with prior evidence indicating that alexithymia—characterized by difficulties in identifying and describing emotions and an externally oriented cognitive style—predisposes individuals to maladaptive emotion regulation strategies, including problematic technology use (39, 40). Specifically, our results revealed that among the alexithymia dimensions, and DIF demonstrated the strongest correlation with PSU, supporting previous research that this component is particularly detrimental to emotional processing and regulation (41).

Theoretically, adolescents with alexithymia may rely on smartphones as a compensatory mechanism to cope with distress, avoid aversive emotional states, and seek immediate gratification through digital interactions (42). Recent theoretical models provide additional insights into the psychological mechanisms underlying this relationship. According to the Compensatory Internet Use Theory (CIUT), individuals with impaired emotion regulation capacities—such as those with alexithymia—are more likely to engage in excessive digital behaviors as a maladaptive strategy to manage negative affect and unmet psychological needs. Given that adolescents with alexithymia struggle to process and verbalize emotions, smartphones offer an immediate, controllable, and socially acceptable outlet for distraction and emotion avoidance (43). Moreover, from the perspective of the I-PACE model (44),core deficits in emotional competence (such as DIF) contribute to the development and maintenance of problematic technology use through reinforcing maladaptive affective responses and promoting habitual engagement with digital media. Specifically, adolescents with alexithymia may experience difficulties interrupting negative affective cycles, leading to repetitive smartphone checking as a form of short-term emotional relief (45). Over time, such compensatory behavior can evolve into compulsive patterns of use, consistent with the high PSU prevalence observed in our sample. A recent study by Lyvers et al. (2022) further confirmed that alexithymia significantly predicts digital overuse across various platforms, including smartphones and social media, particularly in youth populations (46). Moreover, the extremely high prevalence of PSU in adolescents with moderate to high levels of alexithymia observed in our sample (88.7%) underscores the clinical relevance of this association. These findings suggest that alexithymia should be routinely assessed when evaluating risk factors for PSU in adolescents with mood disorders. Furthermore, data collection occurred during the COVID-19 pandemic (2020–2021), which may have intensified social isolation, increased emotional distress, and elevated PSU levels. These contextual influences should be considered when interpreting the results to test the universality of these effects and explore potential cultural moderators (47–49).

### 4.2 Social support as a protective factor: differential effects across sources

One of the key contributions of this study lies in our examination of the moderating role of different sources of social support. Overall, perceived social support was negatively associated with PSU, echoing findings from prior studies (50). However, the protective effects varied significantly by source.

#### 4.2.1 Family support: a robust buffering effect

Family support not only exerted a direct negative association with PSU but also significantly moderated the effect of alexithymia on PSU, suggesting that emotionally supportive family environments may buffer adolescents with poor emotional competence from developing maladaptive digital behaviors. This finding is consistent with family systems theory, which emphasizes the family’s central role in emotion socialization, co-regulation, and internal working model development during adolescence (51). From the lens of attachment theory, adolescents with high family support are more likely to develop secure attachment representations, which in turn foster confidence in expressing emotions and seeking relational support rather than resorting to technological escape (52). Secure family bonds provide a “safe base” for emotion exploration and distress regulation, which may offset the emotional dysregulation associated with alexithymia.

In addition, the emotion socialization model (53), suggests that parents’ emotional responsiveness and validation contribute to the development of children’s emotional awareness and regulation capacity. In families that promote open emotional expression and model adaptive coping, adolescents—even those with trait-level difficulties like alexithymia—may learn to better identify and manage their affect, thereby reducing the need for smartphone-based avoidance strategies (54).

Furthermore, high-quality family support may reduce negative appraisals of stressful situations and promote a sense of belonging and self-worth, which are protective against compulsive smartphone checking (55). In contrast, emotionally disengaged or invalidating family environments may fail to provide the necessary scaffolding, increasing reliance on solitary, screen-based coping methods (56).

Our findings support the view that family support operates not only as an external buffer but also as a developmental context that shapes internal emotional coping schemas. Thus, in adolescents with alexithymia, strong family relationships may serve as a compensatory resilience factor that reduces the risk of developing PSU.

#### 4.2.2 Friend support: complex and context-dependent effects

Friend support also demonstrated a moderating effect on the alexithymia–PSU relationship, although its direct association with PSU was not significant, highlighting the complex and context-dependent nature of peer influences on adolescent digital behaviors.

From a developmental perspective, adolescence is a period when peer relationships assume increasing importance in emotional and social development (57). According to the emotional co-regulation framework (58), supportive friends can serve as vital co-regulators of affect, providing opportunities for emotional validation, perspective-taking, and adaptive coping modeling. For adolescents with alexithymia, who struggle with internal emotional processing, emotionally attuned peer interactions may offer external scaffolding for emotion regulation, thereby reducing reliance on smartphone-based coping strategies (59).

Moreover, friend support can foster a sense of social connectedness and belonging, which is negatively associated with maladaptive digital engagement (60). In this way, positive friend relationships can serve as an alternative source of emotional gratification, reducing the compensatory appeal of smartphones (32).

However, the effect of friend support is likely context-dependent, as peer groups also constitute a potential risk factor for problematic technology use. According to peer contagion theory (61), adolescents often model and reinforce each other’s behaviors. In peer contexts where excessive smartphone use is normalized or even valorized, higher friend support may inadvertently facilitate PSU (62). Additionally, adolescents with alexithymia may gravitate toward online-based friendships, which may lack the protective qualities of in-person peer interactions (Marino et al., 2020), further complicating the role of friend support.

Our findings suggest that the quality and nature of peer relationships are critical in determining whether friend support functions as a protective or risk factor. Interventions targeting adolescent PSU should therefore focus not only on enhancing friend support quantity but also on promoting high-quality, emotionally supportive, and offline peer relationships.

#### 4.2.3 Significant other support: no buffering effect observed

Contrary to expectations, support from significant others did not exhibit a significant moderating effect on the relationship between alexithymia and problematic smartphone use (PSU). This null finding may reflect the unique nature of “significant other” relationships in adolescents with depressive disorders and high alexithymia.

First, the ambiguous and heterogeneous definition of “significant others” in self-report measures—ranging from teachers and coaches to romantic partners or mentors—may contribute to variable psychological relevance. Unlike family and close friends, whose roles are often developmentally primary and emotionally intimate, significant others may occupy peripheral or less consistent positions in the adolescent’s emotional ecology (63). Second, individuals with alexithymia—by definition—struggle with emotional expressiveness, empathy reception, and interpersonal attunement (64). These deficits may impede the ability to accurately perceive or benefit from emotionally nuanced support that significant others typically offer (65). Compared with family members who provide instrumental and unconditional scaffolding, or peers who share symmetrical emotional contexts, significant others may not offer the type of emotionally accessible support that is useful for alexithymic individuals (66). Third, the trust calibration and affective reliance required to benefit from significant other support may be particularly challenging for adolescents with high interpersonal mistrust or emotional constriction—both of which are common among alexithymic and depressive populations (67). As such, the protective potential of these relationships may remain latent unless emotional closeness and perceived responsiveness reach a sufficient threshold. Finally, there is increasing evidence that support perceived as inauthentic or overly distant may not only fail to buffer psychological distress, but may also lead to reactance or disengagement, especially in emotionally dysregulated adolescents (67). For alexithymic individuals, emotional incongruence in significant other interactions may even exacerbate distress and contribute to increased smartphone dependency as a compensatory regulation strategy. Lastly, and importantly, in Chinese culture, family and friends typically play a more significant role in providing emotional support than ‘significant others’ such as romantic partners or mentors, especially during adolescence when these relationships are not yet fully stabilized. Therefore, the lack of a moderating effect of ‘significant other support’ on the relationship between alexithymia and PSU may reflect cultural differences in the roles of emotional support. Overall, these findings suggest that the buffering role of support is highly dependent on the source’s emotional proximity and relational attunement, and that in adolescents with alexithymia, family and close friends may play a more critical protective role than less emotionally embedded figures.

### 4.3 Clinical and theoretical implications

These findings have important implications for both clinical practice and future research. **First**, they emphasize the need for a comprehensive assessment of alexithymia in adolescents with depressive disorders who exhibit problematic smartphone use (PSU) behaviors, especially across different cultural contexts. Given the strong predictive role of alexithymia, particularly the Difficulty Identifying Feelings (DIF) component, interventions aimed at enhancing emotional awareness and expression (e.g., affect labeling, mindfulness-based emotion regulation training) may be especially effective. These interventions can be adjusted to fit various cultural contexts, ensuring they meet the unique emotional and social needs of adolescents in different regions (e.g., family-centered versus peer-centered cultures).Second, our results highlight the protective role of family support, with friend support also playing a significant role to a lesser extent. Specifically, family-based interventions, such as family systems therapy, including multigenerational family sessions and emotion coaching skills training for parents, can enhance parent-child emotional communication and support (68, 69).These interventions can be adjusted to cultural norms to fit the cultural differences in various family structures. Regarding peer support, social skills training and peer support group interventions have been shown to be effective (70, 71), and cultural differences, particularly in how adolescents form and maintain friendships, should be considered. These interventions could be integrated into treatment plans for adolescents at high risk for alexithymia and PSU, especially those from family structures that emphasize peer support over family support. Finally, these findings contribute to refining theoretical models of PSU by emphasizing the interaction between individual emotional traits (e.g., alexithymia) and interpersonal resources (e.g., social support). Research suggests that future theoretical models should account for the differential role of social support across various cultural and familial backgrounds, and how these resources regulate the emotion regulation processes that underpin PSU. Future studies should explore the cross-cultural differences between alexithymia, PSU, and social support, and examine how these findings can inform interventions globally.

## 5 Limitations

Despite the theoretical contributions and practical implications of this study, several limitations should be acknowledged: First, the cross-sectional design precludes causal inferences. Future research should employ longitudinal designs to elucidate the causal pathways between alexithymia and PSU, and could further conduct intervention experiments to evaluate the actual efficacy of enhancing social support in improving adolescents’ smartphone use behaviors. Second, PSU was assessed primarily through self-report questionnaires, which may be subject to social desirability bias and memory distortions. Future research should incorporate objective usage metrics (e.g., smartphone tracking data, application usage logs) to enhance the validity and ecological accuracy of the findings. Third, although the study employed a large, multi-center clinical sample, participants were clinically diagnosed adolescents with depressive disorders. Therefore, caution is warranted when generalizing the findings to non-clinical or community youth populations. Future studies should replicate and extend these findings in more diverse samples. Fourth, the measurement of PSU in this study was relatively global and did not differentiate between specific usage types (e.g., social media, gaming, short video platforms) or usage patterns (e.g., frequency, purpose). Future research should adopt more fine-grained, context-sensitive assessment methods, such as diary techniques and digital behavior tracking, to elucidate distinct behavioral pathways and identify more precise intervention targets. In addition, cultural factors specific to the Chinese adolescent context may have shaped the observed associations. Cross-cultural comparative studies are needed to test the universality of these effects. In addition, Data collection took place during the COVID-19 pandemic (2020–2021), a period that may have intensified adolescents’ feelings of isolation and emotional distress, thereby affecting the patterns of PSU and social support. This contextual factor should be considered when interpreting the results. Finally, the unbalanced gender distribution in our sample, with females comprising 77.9%, may limit the generalizability of the findings, particularly regarding gender-specific behaviors related to depression and smartphone use. This disproportionate representation likely reflects the higher female participation in mental health research, as females generally exhibit greater help-seeking behaviors in clinical settings. However, it is important to note that selection bias cannot be ruled out, especially if certain institutions or regions had a higher proportion of female participants. Therefore, future research should strive for a more balanced gender distribution in sample recruitment to enhance the external validity of the findings. Additionally, future studies should explore the potential moderating effects of other factors (e.g., attachment style, emotion regulation strategies) that may interact with alexithymia and social support in predicting PSU, thereby enriching existing theoretical models and guiding the development of more tailored intervention strategies.

## 6 Conclusion

In conclusion, this study provides robust evidence that alexithymia significantly contributes to PSU in adolescents with depressive disorders and that family and friend support can buffer this risk. These findings underscore the importance of targeting both individual emotional vulnerabilities and social contextual resources in interventions aimed at reducing PSU in this high-risk population.

## Data Availability

The original contributions presented in the study are included in the article/supplementary material. Further inquiries can be directed to the corresponding authors.
